# The role of food addiction in the association between sustainable and healthy eating behaviors and obesity indicators

**DOI:** 10.1007/s00394-026-04039-y

**Published:** 2026-06-29

**Authors:** Jiyan Aslan Ceylan, Çağlar Akçalı, Abdulkerim Hatipoğlu, Aziz Korkmaz, Sedat Coşkunsu

**Affiliations:** 1 Department of Nutrition and Dietetics, Mardin Artuklu University, Diyarbakır Road, 47200 Artuklu, Mardin, Turkey; 2 Present Address: Department of Food Science and Human Nutrition, University of Florida, Gainesville, FL 32606 USA

**Keywords:** Body mass index, Food addiction, Obesity, Sustainable and healthy eating behaviors, Waist circumference

## Abstract

**Purpose:**

This study aimed to examine the association between sustainable and healthy eating behaviors (SHEB) and obesity indicators, body mass index (BMI) and waist circumference (WC), and to evaluate the mediating and moderating roles of food addiction (FA) in these relationships.

**Methods:**

This cross-sectional study was conducted between June 2024 and February 2025 with 4234 adults aged ≥ 18 years. Anthropometric measurements were obtained using standardized procedures. SHEB and FA were assessed using validated instruments. All analyses were conducted using the SPSS package program, PROCESS Macro and JASP Statistical Software.

**Results:**

In adjusted models, each one-unit increase in FA symptom count was positively associated with BMI (β = 0.103, *p* < 0.001) and WC (β = 0.030, *p* = 0.029). Overall SHEB scores showed a small positive association with BMI, whereas no association was observed with WC. SHEB subcomponents related to diet quality, including healthy and balanced diet, preference for local foods, and low-fat choices, were inversely associated with both BMI and WC (*p* < 0.05), whereas seasonal food consumption and avoidance of food waste were positively associated with obesity indicators. FA showed a statistically significant but very small indirect effect in the association between SHEB and BMI (indirect effect = 0.0004; 95% CI: 0.000–0.001), while no indirect effect was observed for WC. Moderation analyses revealed that FA significantly modified the SHEB-BMI relationship (*p* for interaction = 0.003), with stronger associations observed at moderate and high FA levels.

**Conclusions:**

Sustainable and healthy eating behaviors are not uniformly associated with lower obesity risk and appear to interact with FA-related behaviors. Accounting for addictive-like eating behaviors may be critical for improving the effectiveness of sustainable nutrition strategies in obesity prevention and public health interventions.

**Supplementary Information:**

The online version contains supplementary material available at 10.1007/s00394-026-04039-y.

## Introduction

Obesity is one of the most significant public health challenges worldwide, with a steadily increasing prevalence and serious adverse health consequences. Overweight and obesity are among the leading modifiable risk factors for numerous chronic conditions, including type 2 diabetes, cardiovascular diseases, and several cancers [1]. Although BMI is widely used to assess general adiposity, WC provides complementary information by capturing abdominal obesity and visceral fat accumulation, which are more strongly linked to cardiometabolic risk [2].

As the global burden of obesity continues to rise, interest in dietary models that support both human health and environmental sustainability have increased. International guidelines and policy frameworks emphasize dietary patterns characterized by higher consumption of plant-based foods, reduced intake of animal-source foods, and decreased food waste as key strategies to improve population health while reducing environmental impact [3, 4]. Within this framework, SHEB have emerged as a multidimensional construct encompassing nutritional adequacy alongside environmental responsibility. These behaviors include maintaining a healthy and balanced diet, reducing meat intake, prioritizing local and seasonal foods, choosing low-fat options, and avoiding food waste [4, 5]. Given their association with diets lower in energy density and higher in fiber, SHEB are often presumed to be protective against excessive weight gain and adiposity [6].

While some studies report a reduced risk of obesity associated with sustainable dietary practices [7], others indicate weak or statistically insignificant relationships [5, 6]. These mixed findings suggest that the relationship between SHEB and obesity may be influenced not only by dietary patterns themselves but also by individual behavioral and psychological factors. From this perspective, the concept of FA has attracted increasing research attention.

SHEB are generally expected to align with healthier dietary profiles and lower levels of FA symptoms. Previous studies have reported that individuals with higher FA symptoms often exhibit lower adherence to SHEB [8]. However, this relationship may be more complex than traditionally assumed. Research suggests that foods perceived as “healthy,” “natural,” or “sustainable” can influence individuals’ consumption perceptions and eating behaviors [9, 10]. Similarly, moral licensing theory proposes that engaging in behaviors perceived as virtuous or environmentally responsible may psychologically justify subsequent pleasure-seeking eating behaviors [11]. Collectively, these findings suggest that the relationship between SHEB and FA symptoms may involve heterogeneous and potentially counterintuitive behavioral mechanisms.

FA is derived from the diagnostic criteria for substance use disorders and is characterized by loss of control over eating, continued consumption despite negative consequences, and compulsive intake of highly processed, energy-dense foods [12, 13]. Clinical and epidemiological evidence shows that FA symptoms are associated with higher energy intake, disordered and binge eating behaviors, as well as increased BMI and WC [14, 15]. Studies using the Yale Food Addiction Scale (YFAS) consistently report greater eating-related psychopathology, including binge eating and loss-of-control eating, among individuals with higher FA symptom severity [16, 17]. Moreover, experimental and neurobiological research indicates that highly processed foods influence reward-related and inhibitory control mechanisms as well as energy intake regulation, thereby contributing to excessive weight gain [18]. Collectively, these findings suggest that FA may be relevant to understanding the associations between dietary patterns and obesity-related outcomes.

Despite these insights, it remains unclear whether FA explains (mediates) the association between SHEB and obesity indicators or whether FA alters (moderates) the strength of this relationship across different levels of addictive-like eating behavior. Therefore, the aim of the present study was to examine the associations between SHEB and obesity indicators (BMI and WC) in a large community-based adult sample and to evaluate the potential mediating and moderating roles of FA in these relationships. Accordingly, the following hypotheses were tested: (1) higher adherence to SHEB would be associated with lower BMI and WC; (2) higher levels of FA symptoms would be positively associated with BMI and WC; (3) FA would mediate the relationship between SHEB and obesity indicators; and (4) FA would function as a moderating variable, altering the strength or direction of the association between SHEB and obesity indicators. Elucidating these pathways may contribute to a clearer public health–relevant understanding of how SHEB relate to obesity indicators through behavioral mechanisms such as FA.

## Methods

### Study design and participants

This cross-sectional study was conducted between June 2024 and February 2025 in Mardin, Türkiye, and comprised adults recruited via a convenience sampling strategy from diverse community settings, including educational institutions, public areas, and health centers. Eligible participants were adults aged 18 years and older who provided informed consent. Individuals were excluded if they had extreme BMI values (< 16.0 or > 45.0 kg/m^2^, n = 160) to minimize the potential influence of clinically extreme anthropometric measurements and atypical eating-related conditions on regression estimates. Individuals were also excluded if they had a current or past diagnosis of eating disorders, or severe neurological or psychiatric conditions that could affect eating behavior or cognitive functioning (e.g., schizophrenia, bipolar disorder) (n:126). Participants were additionally excluded if they were pregnant or had given birth within the past two years (n:100), or had serious chronic conditions likely to affect body composition or dietary behavior, including end-stage renal or hepatic disease, active malignancy, or clinically significant gastrointestinal disorders (n:300), or had used nutritional supplements or followed a medically prescribed or self-initiated therapeutic diet (e.g., weight-loss, diabetes, low-fat, low-sodium, or low-cholesterol diets) within the previous six months (n:290). Participants meeting one or more exclusion criteria were excluded from the study, and the reported numbers reflect unique excluded individuals. Of the 5210 individuals initially assessed for eligibility, 4234 met the study criteria and were enrolled in the analyses (Supplementary Figure S1).

The study protocol was approved by the Ethics Committee of Mardin Artuklu University (decision no: 2024/6–27, 11 June 2024), and all procedures adhered to the ethical guidelines outlined in the Declaration of Helsinki.

### Data collection and instruments

Data were collected by trained researchers using a structured, face-to-face interview method. After obtaining written informed consent, participants completed a comprehensive questionnaire that included sociodemographic characteristics (age, gender, education level, occupation), lifestyle factors (smoking status, alcohol consumption, and physical activity), health status (presence of chronic diseases), anthropometric measurements (weight, height, WC), SHEB and FA scales. Chronic disease status was assessed based on participants’ self-reported medical history and coded as a binary variable (yes/no) indicating the presence of at least one chronic disease including hypertension, diabetes, hyperlipidemia, respiratory diseases (e.g., asthma, chronic bronchitis), gastrointestinal diseases (e.g., gastritis, peptic ulcer), and musculoskeletal disorders (e.g., arthritis, osteoporosis). Physical activity was assessed using a self-reported question regarding the frequency of engaging in approximately 30 min of moderate-intensity activities that cause a slight increase in heart rate (e.g., brisk walking or light cycling). Participants were categorized as none, 1–2 days/week, 3–4 days/week, or ≥ 5 days/week according to their reported frequency of moderate-intensity physical activity.

Anthropometric measurements were obtained by the researchers according to standardized protocols. To ensure data quality, all researchers received prior training on questionnaire administration, interview techniques, and anthropometric measurement procedures. Body weight (kg) was measured to the nearest 0.1 kg using a calibrated digital scale with participants wearing light clothing and no shoes. Height (cm) was measured to the nearest 0.1 cm using a portable stadiometer, and BMI was calculated as weight in kilograms divided by height in meters squared (kg/m^2^). Participants were categorized according to BMI into two groups: < 25 kg/m^2^ (normal weight) and ≥ 25 kg/m^2^ (overweight/obese), in accordance with World Health Organization cut-off values [1]. WC was measured at the midpoint between the lowest rib and the iliac crest at the end of a normal expiration using a non-elastic measuring tape. Measurements were performed in duplicate, and the average value was used in the analyses. WC data were unavailable for 277 participants; therefore, analyses involving WC were conducted using the available data.

### Sustainable and healthy eating behaviors

SHEB were assessed using the Sustainable and Healthy Eating Behaviors Scale, which was adapted into Turkish by Köksal et al. [19] from the original scale by Żakowska-Biemans et al. [20]. It consists of seven factors and 32 items, including quality labels, seasonal foods and avoiding food waste, animal welfare, reducing meat consumption, healthy and balanced nutrition, preferring local foods, and choosing low-fat foods. Items are rated on a 7-point Likert scale (1 = never, 7 = always), with higher scores reflecting greater adherence to sustainable and health-promoting dietary behaviors. Factor scores were calculated by averaging the scores given to the items in the relevant factor (1–7 points), and the total scale score was calculated by averaging the scores given to these factors. In this study, the Cronbach’s alpha of the scale was 0.945.

### Yale food addiction scale

FA was evaluated using the Yale Food Addiction Scale, an instrument grounded in the DSM-IV substance dependence framework and developed to assess addictive-like eating behaviors [13]. The Turkish-adapted and psychometrically validated version of the scale was employed in the present study [21]. The YFAS consists of 25 items representing seven dependence-related symptom domains adapted to eating behavior: excessive time devoted to food-related activities, consuming larger amounts than intended, continued consumption despite negative consequences, tolerance, repeated unsuccessful attempts to reduce intake, neglect of social or occupational roles, and withdrawal-like symptoms. The scale assesses food addiction symptoms occurring during the previous 12 months, consistent with the DSM-IV substance dependence framework. Items are rated on Likert-type frequency scales, and each symptom domain is coded as present or absent according to established scoring thresholds. Two outcome measures were derived. First, a continuous symptom count (range: 0–7) was calculated by summing the number of endorsed symptom domains, with higher scores indicating greater severity of addictive-like eating. Second, a categorical classification was generated, whereby individuals meeting three or more symptom criteria were considered to exhibit FA, consistent with the standard YFAS scoring approach. The scale demonstrated very high internal reliability in the present sample (Cronbach’s α = 0.913).

### Statistical analysis

All analyses were conducted using the Statistical Package for the Social Sciences (SPSS) package program (IBM SPSS Statistics 29.0.2.0. Armonk, NY, USA: IBM Corp; 2024), PROCESS Macro [22] and JASP Statistical Software (version 0.18.2, Netherlands). Descriptive statistics were computed for all variables. Continuous variables were presented as mean and standard deviation (SD), while categorical variables were presented as frequencies and percentages. Normality was evaluated via histograms, Q–Q plots, skewness and kurtosis.

Group comparisons according to BMI categories were conducted using chi-square tests for categorical variables and independent samples t-tests for continuous variables. Effect sizes were calculated as Phi coefficient for 2 × 2 tables, and Cramer’s V for larger contingency tables and Cohen’s d for t-tests. Bivariate associations among variables were evaluated using Pearson correlation coefficients with two-tailed tests. All variables were treated as continuous variables. For visualization, bivariate scatter plots were created with ordinary least squares linear fits and 95% confidence intervals.

Multiple linear regression analyses were performed to investigate the independent associations of SHEB dimensions and FA with BMI and WC. Standardized regression coefficients (β), unstandardized coefficients (B) with 95% confidence intervals, R^2^ values, and F statistics were reported. All models were adjusted for age, gender, education level, smoking status, presence of chronic disease, and physical activity. Multicollinearity was assessed using variance inflation factors (VIF), and all values were below the acceptable threshold (< 5).

Mediation analyses were conducted using PROCESS macro-Model 4 without the inclusion of covariates to test whether FA mediated the relationship between SHEB and BMI and WC. Indirect effects were estimated using bias-corrected bootstrap confidence intervals based on 5000 resamples. Mediation was considered significant if the 95% CI excluded zero. Moderation analyses were performed using PROCESS macro-Model 1 without the inclusion of covariates to examine whether FA moderated the association between SHEB and obesity indicators. All continuous variables were mean-centered prior to creating the interaction term to reduce multicollinearity and improve interpretability. Interaction terms were included and model assumptions of linearity, homoscedasticity, and error independence were verified prior to hypothesis testing. Conditional effects were probed at the 16th, 50th, and 84th percentiles of the moderator, corresponding to low, median, and high levels, as recommended in the PROCESS macro approach for probing interaction effects, providing a more robust and distribution-based alternative to ± 1 SD. Statistical significance was set at *p* < 0.05 throughout.

## Results

In the analytic sample (N = 4234), 37.0% of participants were overweight/obese (BMI ≥ 25 kg/m^2^). Compared with those with BMI < 25 kg/m^2^, participants in the overweight/obese group were older, more often male, had lower educational attainment, and more likely to be employed (*p* < 0.05). Chronic disease prevalence was higher and physical activity patterns differed significantly in the overweight/obese group (*p* < 0.05), whereas smoking status and alcohol consumption did not differ significantly between BMI categories. Mean SHEB scores were slightly but significantly higher in the BMI ≥ 25 kg/m^2^ group (3.7 ± 1.0 vs 3.6 ± 1.0; *p* < 0.001; d = 0.11). FA symptom counts were marginally higher among participants with BMI ≥ 25 kg/m^2^ (3.1 ± 1.7 vs 2.9 ± 1.7; *p* < 0.001; d = 0.11), whereas the prevalence of categorical FA did not differ (36.5% vs 34.3%; *p* = 0.142) (Table 1).

**Table 1:** Participant characteristics by BMI category (N = 4234)

Characteristic	< 25 kg/m^2^ (n = 2669)	≥ 25 kg/m^2^ (n = 1565)	*P-*value	Effect size
Age (years), M ± SD	26.7 ± 7.8	35.6 ± 10.9	< 0.001^a^	0.985
Age group, n (%)			< 0.001^b^	0.434
18–24	1445 (54.1)	284 (18.2)		
25–34	856 (32.1)	507 (32.4)		
35–44	242 (9.1)	395 (25.2)		
45–64	126 (4.7)	379 (24.2)		
Gender, n (%)			< 0.001^b^	0.133
Male	951 (35.6)	770 (49.2)		
Female	1718 (64.4)	795 (50.8)		
Education level, n (%)			< 0.001^b^	0.307
Primary school	233 (8.7)	505 (32.2)		
High school	1305 (48.9)	655 (41.9)		
University or higher	1131 (42.4)	405 (25.9)		
Occupation, n (%)			< 0.001^b^	0.318
Not employed / Homemaker	541 (20.3)	617 (39.4)		
Student	1212 (45.4)	237 (15.2)		
Employed	916 (34.3)	711 (45.4)		
Chronic disease, n (%)			< 0.001^b^	0.161
No	2258 (84.6)	1,114 (71.2)		
Yes	411 (15.4)	451 (28.8)		
Current smoker, n (%)			0.277^b^	0.017
Yes	987 (37.0)	605 (38.7)		
No	1682 (63.0)	960 (61.3)		
Alcohol consumption, n (%)			0.436^b^	0.012
Yes	430 (16.1)	238 (15.2)		
No	2239 (83.9)	1327 (84.8)		
Physical activity, n (%)			0.031^b^	0.046
≥ 5 days/week	233 (8.7)	126 (8.0)		
3–4 days/week	492 (18.5)	347 (22.2)		
1–2 days/week	934 (35.0)	518 (33.1)		
None	1010 (37.8)	574 (36.7)		
WC (cm), M ± SD	71.9 ± 10.2	87.7 ± 11.8	< 0.001^a^	1.462
SHEB, M ± SD	3.6 ± 1.0	3.7 ± 1.0	< 0.001^a^	0.108
FA symptom count, M ± SD	2.9 ± 1.7	3.1 ± 1.7	< 0.001^a^	0.107
Food addicted, n (%)	916 (34.3)	572 (36.5)	0.142^b^	0.023
Non-food addicted, n (%)	1753 (65.7)	993 (63.5)		

The values are presented as the means ± SDs or n (%). ^a^ t-test; ^b^ χ2 test. p < 0.05 was considered statistically significant. Effect size: Phi (2 × 2 chi-square), Cram´er’s V (larger chi-square tables), Cohen’s d (independent samples t-test). SHEB, sustainable and healthy eating behaviors; FA, food addiction; BMI, body mass index; WC, waist circumference

**Fig. 1:**
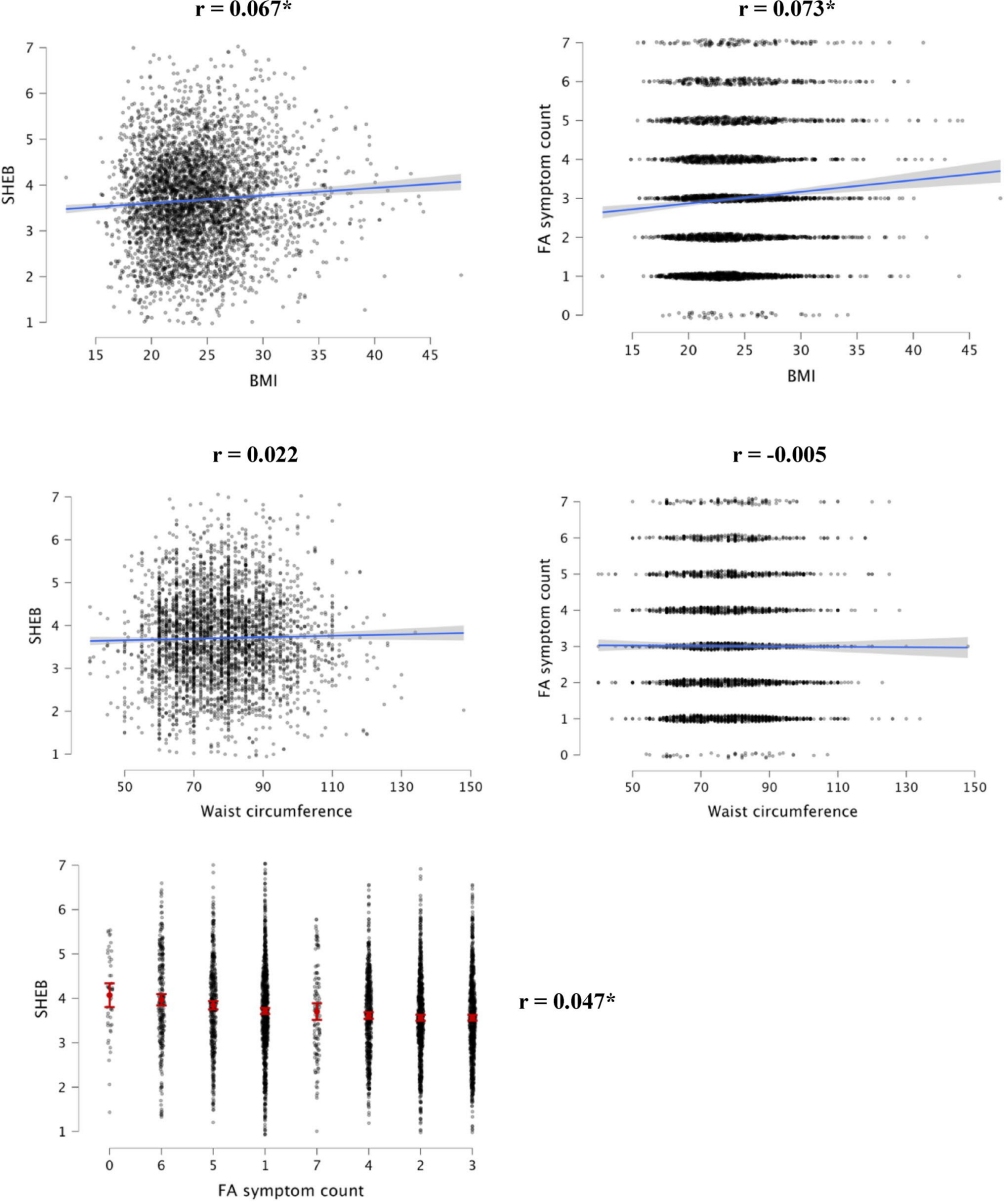
Bivariate scatterplots among study variables. Each panel shows individual data points with a linear fit and 95% confidence band. Pearson correlations are two-tailed; asterisks denote *p* < 0.05

Bivariate scatterplots showed statistically significant but trivial positive correlations between SHEB and BMI (r = 0.067, *p* < 0.001), whereas no association was observed with WC (r = 0.022, *p* = 0.162). FA symptom counts were likewise trivially correlated with BMI (r = 0.073, *p* < 0.001) and were unrelated to WC (r = -0.005, *p* = 0.777). The correlation between SHEB and YFAS symptom counts was very small (r = 0.047, *p* < 0.001) (Fig. 1).

In multivariable models adjusted for age, gender, education level, smoking status, presence of chronic disease, and physical activity, several SHEB components were independently associated with obesity indicators. For BMI, adherence to healthy and balanced diet (β =  − 0.104, *p* < 0.001), preference for local foods (β =  − 0.050, *p* = 0.006), and low-fat choices (β =  − 0.054, *p* = 0.004) were associated with lower BMI, whereas greater use of quality labels and higher scores on seasonal food consumption/avoiding food waste showed small positive associations (β = 0.089 and 0.094 respectively; *p* < 0.001). For WC, meat reduction, healthy and balanced diet, local food preference, and low-fat choices were associated with lower WC (β range =  − 0.088 to − 0.049, *p* < 0.05), whereas seasonal food consumption/avoiding food waste and animal welfare concerns were associated with higher WC (β = 0.100 and 0.063, respectively; *p* < 0.05). In adjusted models, FA symptoms were positively related to both BMI (β = 0.103, *p* < 0.001) and WC (β = 0.032, *p* = 0.029). The overall models explained 28.6% of the variance in BMI and 29.9% of the variance in WC (Table 2).

**Table 2:** Associations of sustainable and healthy eating behaviors factors and food addiction with BMI and waist circumference

Predictor	BMI			WC		
	B (95% CI)	Standardized β	*p*-value	B (95% CI)	Standardized β	*p*-value
Quality labels	0.04 (0.02, 0.06)	0.089	< 0.001	0.05 (− 0.00, 0.11)	0.035	0.069
Seasonal food and avoiding food waste	0.05 (0.03, 0.07)	0.094	< 0.001	0.16 (0.09, 0.22)	0.100	< 0.001
Animal welfare	0.02 (− 0.01, 0.05)	0.028	0.159	0.15 (0.05, 0.24)	0.063	0.002
Meat reduction	0.02 (− 0.02, 0.05)	0.015	0.412	− 0.16 (− 0.28, − 0.04)	− 0.050	0.009
Healthy and balanced diet	− 0.08 (− 0.11, − 0.05)	− 0.104	< 0.001	− 0.14 (− 0.25, − 0.04)	− 0.058	0.007
Local food	− 0.05 (− 0.08, − 0.01)	− 0.050	0.006	− 0.26 (− 0.37, − 0.15)	− 0.088	< 0.001
Low fat	− 0.05 (− 0.08, − 0.02)	− 0.054	0.004	− 0.14 (− 0.25, − 0.03)	− 0.049	0.011
Food addiction symptoms	0.25 (0.19, 0.32)	0.103	< 0.001	0.23 (0.02, 0.44)	0.030	0.029
Model fit	R^2^ = 0.286, F(14, 4219) = 120.97, *p* < 0.001			R^2^ = 0.299, F(14,3942) = 120.17, *p* < 0.001		

Values are standardized regression coefficients (β) and unstandardized coefficients (B) with 95% confidence intervals from multiple linear regression. Models were adjusted for age, gender, education level, smoking status, presence of chronic disease, and physical activity. BMI, body mass index; WC, waist circumference. *p* < 0.05 was considered statistically significant

**Fig. 2:**
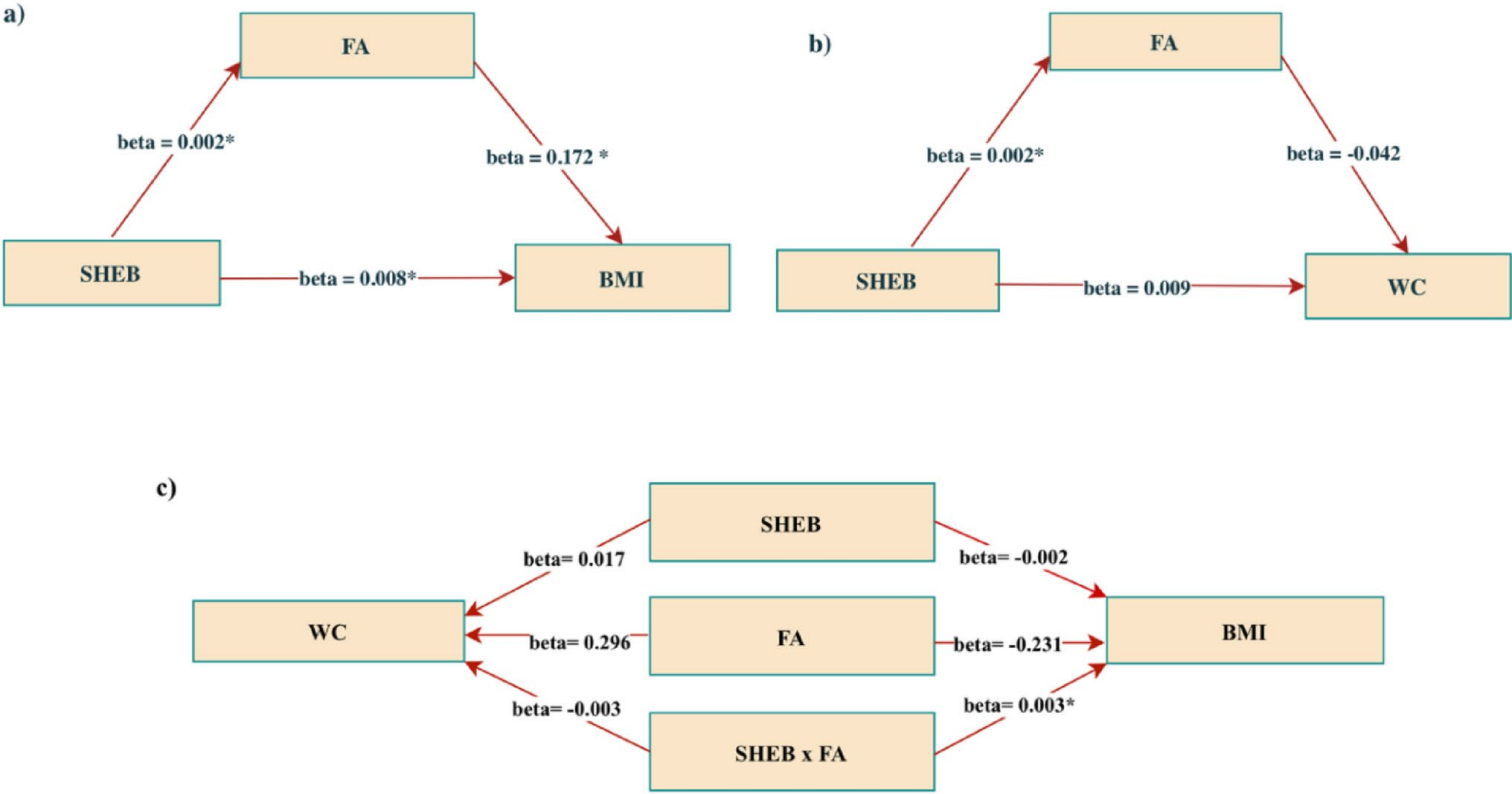
Mediation and moderation models examining the relationships among sustainable and healthy eating behaviors (SHEB), food addiction (FA), and obesity indicators. Panel **a** illustrates the mediation model in which FA mediates the association between SHEB and body mass index (BMI). Panel **b** shows the mediation model for waist circumference (WC). Panel **c** presents the moderated regression model testing the interactive effect of SHEB and FA (SHEB × FA) on BMI and WC. Asterisks denote statistically significant paths (*p < 0.05)

FA was examined as a potential mediator in the association between SHEB and obesity indicators (Table 3, Fig. 2a, b). For BMI, higher SHEB scores were positively associated with FA (path a: B = 0.002, *p* = 0.002). FA, in turn, was positively associated with BMI (path b: B = 0.172, *p* < 0.001). The direct effect of SHEB on BMI remained significant (path c′: B = 0.008, *p* < 0.001), and the indirect effect through FA was small but statistically significant (a × b = 0.0004, 95% CI = 0.000–0.001), indicating partial mediation. For WC, SHEB was also positively associated with FA (path a: B = 0.002, *p* = 0.014); however, FA was not associated with WC (path b: B =  − 0.042, *p* = 0.735). Neither the indirect effect (a × b = -0.0001, 95% CI =  − 0.001–0.000) nor the direct effect of SHEB on WC (path c′: B = 0.009, *p* = 0.159) reached statistical significance.

**Table 3:** Mediation of food addiction in the association between sustainable and healthy eating behaviors and obesity indicators

Path	B (SE)	β	t	*p*	95% CI (Lower–Upper)
Body mass index (BMI)					
SHEB → FA (a)	0.002 (0.001)	0.047	3.05	0.002	0.001–0.004
FA → BMI (b)	0.172 (0.038)	0.070	4.59	< 0.001	0.099–0.246
SHEB → BMI (direct, c′)	0.008 (0.002)	0.064	4.19	< 0.001	0.004–0.012
Indirect effect (a × b)	0.0004 (BootSE = 0.0002)	0.003			0.000–0.001
Waist circumference (WC)					
SHEB → FA (a)	0.002 (0.001)	0.039	2.46	0.014	0.000–0.004
FA → WC (b)	− 0.042 (0.123)	− 0.005	− 0.34	0.735	− 0.283–0.200
SHEB → WC (direct, c′)	0.009 (0.006)	0.022	1.41	0.159	− 0.004–0.022
Indirect effect (a × b)	− 0.0001 (BootSE = 0.0003)	− 0.000			− 0.001–0.000

Unstandardized coefficients (B) with standard errors (SE) and standardized coefficients (β) are reported. Indirect effects were estimated using bias-corrected bootstrap confidence intervals based on 5000 resamples. SHEB = sustainable and healthy eating behaviors; FA, food addiction; BMI, body mass index; WC, waist circumference

The moderation analysis showed that for BMI, the interaction term (SHEB × FA) was significant (B = 0.003, *p* = 0.003), suggesting that the association between SHEB and BMI varies by FA level. Conditional effects analysis revealed that SHEB was not associated with BMI at low levels of FA (B = 0.002, *p* = 0.600), yet the association became significant at median (B = 0.008, *p* < 0.001) and high levels of FA (B = 0.015, *p* < 0.001), indicating that the positive relationship between SHEB and BMI strengthens as FA increases. In contrast, for WC, neither the main effect of SHEB (B = 0.017, *p* = 0.168) nor the interaction term (SHEB × FA; B = -0.003, *p* = 0.443) was statistically significant, indicating no moderating effect of FA on the SHEB-WC relationship (Table 4, Fig. 2c).

**Table 4:** Moderation of food addiction on the association between sustainable and healthy eating behaviors and obesity indicators

Predictor	B (SE)	β	t	*p*	95% CI (Lower–Upper)
Waist circumference (WC)					
SHEB (X)	0.017 (0.013)	0.022	1.38	0.168	− 0.007–0.042
FA (W)	0.296 (0.458)	0.014	0.65	0.517	− 0.601–1.193
SHEB × FA (Int_1)	− 0.003 (0.004)	− 0.007	− 0.77	0.443	− 0.010–0.004
Body mass index (BMI)					
SHEB (X)	− 0.002 (0.004)	− 0.005	− 0.47	0.638	− 0.009–0.006
FA (W)	− 0.231 (0.139)	− 0.021	− 1.66	0.097	-0.504–0.042
SHEB × FA (Int_1)	0.003 (0.001)	0.046	3.01	0.003	0.001–0.006

FA level (percentile)	Effect of SHEB on BMI	SE	t	*p*	95% CI (Lower–Upper)
Conditional effects of SHEB on BMI at different levels of FA					
16th percentile (low)	0.002	0.003	0.52	0.600	− 0.004–0.007
50th percentile (median)	0.008	0.002	4.23	< 0.001	0.004–0.012
84th percentile (high)	0.015	0.003	5.02	< 0.001	0.009–0.021

B, unstandardized coefficient; SE, standard error; β, standardized coefficient. Conditional effects calculated at 16th (low), 50th (median), and 84th (high) percentiles of FA. SHEB = sustainable and healthy eating behaviors; FA, food addiction; BMI, body mass index; WC, waist circumference

## Discussion

The present study examined the associations between SHEB and obesity indicators, namely BMI and WC, and evaluated the mediating and moderating roles of FA in these relationships. Contrary to our initial hypothesis, higher overall SHEB scores were not consistently associated with lower obesity indicators. Instead, the total SHEB score showed a very small positive association with BMI and no significant association with WC. These findings suggest that SHEB, when assessed as an overall score, should not be interpreted as a uniform protective dietary pattern in relation to obesity indicators.

The very small positive association between the total SHEB score and BMI should be interpreted cautiously. Although statistically significant, the magnitude of this association was small and may partly reflect the large sample size. More importantly, the total SHEB score combines several heterogeneous behavioral dimensions, including diet-quality-related practices, environmental concerns, local and seasonal food preferences, animal welfare considerations, meat reduction, and avoidance of food waste. These dimensions may not operate in the same direction in relation to body weight. Therefore, the positive association observed for the total score should not be interpreted as evidence that sustainable eating as a whole increases BMI. Rather, it indicates that the overall SHEB score may mask divergent associations across its subcomponents.

The subcomponent-level analyses further support the interpretation that SHEB is a multidimensional construct with heterogeneous associations with obesity indicators. These findings are broadly consistent with previous studies suggesting that diet-quality-related dimensions of sustainable eating may be more relevant to obesity indicators than sustainability-oriented behaviors alone [18–21]. However, the finding that sustainability-oriented components such as seasonal food consumption, avoiding food waste and paying attention to quality labels were positively associated with BMI and especially WC suggests that SHEB may not necessarily correspond with lower energy intake or lower adiposity [7, 24, 25]. The positive associations observed for some sustainability-oriented components should be interpreted with caution. Because this study did not assess total energy intake, portion size, perceived healthiness of foods, or food energy density, mechanisms related to overconsumption cannot be directly inferred from the present data. One possible explanation is that participants with higher BMI may have become more attentive to sustainable or health-related eating behaviors after weight gain, suggesting potential reverse causation. Another possibility is social desirability bias, as individuals may over-report behaviors perceived as healthy, ethical, or environmentally responsible. In addition, residual confounding by socioeconomic position, food purchasing capacity, local food availability, cultural dietary practices, or unmeasured lifestyle characteristics may have influenced these associations. Therefore, these findings should be regarded as hypothesis-generating rather than causal.

The positive association between quality-label use and obesity indicators also requires cautious interpretation. Paying attention to quality labels may reflect greater health or sustainability awareness; however, it does not necessarily imply lower energy intake, better dietary composition, or healthier food choices in all contexts. Quality-labelled foods may vary considerably in nutritional profile, and some may still be energy-dense. In addition, individuals with higher BMI may be more likely to report attention to quality labels because of increased health consciousness following weight gain, which raises the possibility of reverse causation. Residual confounding by socioeconomic status, purchasing patterns, food availability, or other lifestyle factors may also partly explain this association.

In multiple linear regression analysis, FA symptoms were positively associated with both BMI and WC, supporting the effect of psychological and behavioral factors on obesity [26–28]. It is suggested that FA symptoms may lead to compulsive eating behaviors by weakening self-control even in individuals with healthy eating intentions [29]. The partial mediation and moderation findings suggest that FA symptoms may influence how SHEB relate to BMI. However, given the statistically significant but very small positive association between the total SHEB score and BMI, these results should not be interpreted as evidence that higher overall SHEB has a protective effect on body weight in the present sample. These findings suggest that the relationship between SHEB and obesity is not unidimensional and is shaped by psychological factors, especially FA symptoms. The impact of sustainable nutrition on obesity should be assessed not only by dietary content but also by accompanying psychological processes. Therefore, interventions for obesity should include integrated approaches including FA symptoms and self-control processes, which is important for public health by supporting behavior change and metabolic risk reduction.

Mediation analyses indicated that FA statistically mediated the association between SHEB and BMI; however, the magnitude of the indirect effect was extremely small. Therefore, although the indirect pathway reached statistical significance, probably due in part to the large sample size, it should not be interpreted as clinically meaningful at the individual level. This finding suggests that FA may be statistically detectable as part of the SHEB-BMI pathway, but its explanatory contribution is limited. Accordingly, the mediation result should be interpreted cautiously and should not be overextended as evidence of a clinically important mechanism without confirmation from longitudinal or intervention studies. This result is consistent with the literature suggesting that the relationship between dietary behaviors and obesity is shaped not only by diet quality but also by psychological characteristics [30, 31]. Moreover, it has been reported that FA is associated with self-esteem and emotional eating patterns, and these characteristics may facilitate weight gain [32, 33]. In this context, the mediating role of FA in the association between SHEB and BMI suggests that addictive-like eating behaviors may undermine weight control efforts, even among individuals with intentions to adopt healthy eating behaviors. Therefore, psychological mechanisms such as FA should also be considered in sustainable nutrition interventions.

In the study, although a positive relationship was found between SHEB and FA, the effect of FA on WC was not statistically significant. This finding is consistent with previous evidence suggesting that although BMI and WC are related indicators, they reflect different biological and physiological dimensions of obesity [34]. Some studies suggest that psychological variables exhibit stronger associations with BMI, while WC may be shaped by hormonal, metabolic and genetic mechanisms [35, 36]. Therefore, the lack of a significant effect of FA on WC can be considered as a reflection of the multidimensional nature of obesity.

Importantly, moderation analyses revealed that FA altered the strength of the SHEB–BMI association. The absence of a significant association at low FA levels and the progressively stronger relationship at moderate and high FA levels indicate that FA may act as a contextual factor shaping how sustainable eating behaviors relate to body weight. This finding suggests that the effects of psychological factors on obesity are not constant and FA may function as a critical variable. Therefore, targeting not only dietary behaviors but also psychological regulators such as FA symptoms and self-esteem in obesity interventions may provide more effective results.

One of the main strengths of this study is that it examined the associations between SHEB, obesity indicators (BMI and WC) and FA in a large, community-based sample of adults in a holistic and theoretical framework. The combined assessment of BMI and WC provides a broader perspective by distinguishing between relative body weight and abdominal adiposity. Measuring SHEB and FA with valid and reliable scales, collecting anthropometric measurements objectively by researchers using standardized protocols, and controlling for key sociodemographic and lifestyle variables in the analyses increase the methodological robustness of the findings. Furthermore, mediation and moderation analyses using bootstrap confidence intervals revealed direct and indirect effects of the SHEB-obesity relationship.

However, some limitations should be considered. First, the cross-sectional design limits inferences about causality between variables. Although anthropometric measurements were objectively obtained, self-report-based assessment of behavioral and psychological variables may lead to reporting and social desirability bias. In addition, physical activity was assessed using a brief self-reported frequency-based measure, which may not have fully reflected all dimensions of physical activity behavior. Chronic disease status was based on self-reported medical history, and the potential effects of different chronic conditions could not be evaluated in detail. The findings can only be generalized to the general population with sociodemographic characteristics similar to those of the study sample. The fact that the study was conducted in a single province and that the socioeconomic structure has regional characteristics may limit the direct transfer of the results to different regional contexts.

## Conclusions

This study showed that the relationship between SHEB and obesity indicators was complex and could not be adequately explained by the total SHEB score alone. Contrary to the initial hypothesis, higher overall SHEB was not consistently associated with lower BMI or WC. Instead, different SHEB components showed heterogeneous associations with obesity indicators, suggesting that health-oriented, environmental, ethical, and consumer-related dimensions of sustainable eating should be considered separately. FA symptoms were independently associated with BMI and WC in adjusted models. Although FA statistically mediated the association between SHEB and BMI, the indirect effect was extremely small and should not be interpreted as clinically meaningful. FA also moderated the SHEB–BMI association, indicating that addictive-like eating symptoms may influence how SHEB relate to body weight. No mediation or moderation effect was observed for WC. Overall, these findings suggest that sustainable nutrition strategies should not rely solely on broad behavioral scores, but should also consider specific dietary dimensions and psychosocial factors such as addictive-like eating. Longitudinal and intervention studies are needed to clarify causal pathways and to determine whether addressing FA can improve the effectiveness of sustainable and healthy eating interventions in obesity prevention.

## Supplementary Information

Below is the link to the electronic supplementary material.

Supplementary Material 1

## Data Availability

The datasets used during the current study are available from the corresponding author upon reasonable request.
