# Supplementary material for: The role of food addiction in the association between sustainable and healthy eating behaviors and obesity indicators
Source: Eur J Nutr. 2026 Jun 29;65(5):178. doi: 10.1007/s00394-026-04039-y (PMC13315278; doi:10.1007/s00394-026-04039-y)
Supplement: Supplementary file 1 — Supplementary Material 1 [file 394_2026_4039_MOESM1_ESM.docx]

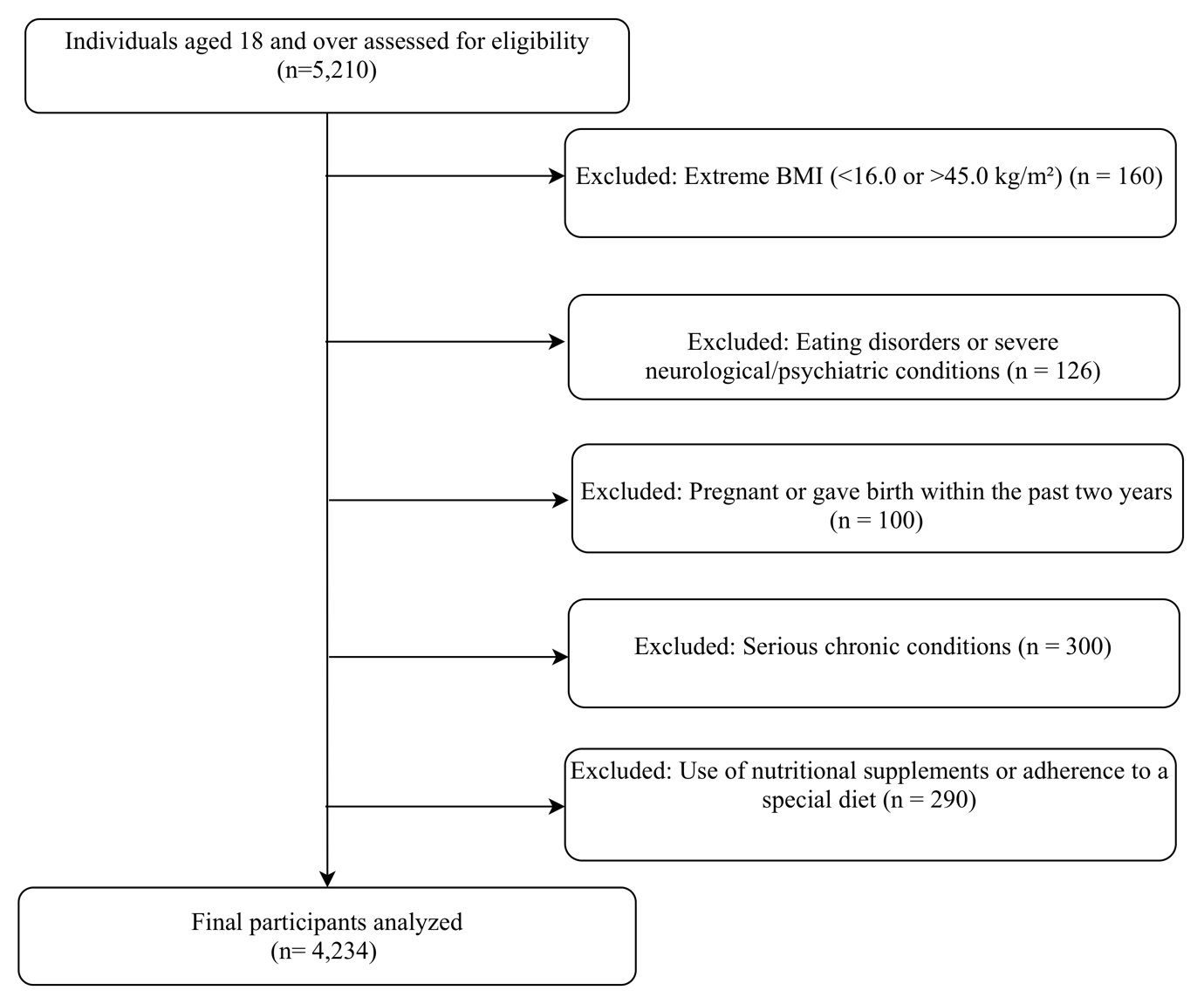


**Supplementary Figure S1.** Participant flow diagram. *Note:* Participants meeting one or more exclusion criteria were excluded. The numbers shown represent unique individuals assigned to a single exclusion category for reporting purposes.
